# Epidemiology of Urban Malaria in the Greater Accra Region of Ghana: Predictors and Effect Modification across a Transmission Season

**DOI:** 10.4269/ajtmh.26-0325

**Published:** 2026-10-27

**Authors:** Michaela McGuire, Anna Beltrame, Francis Dzabeng, Victor Edem Kornu, Clinton N. Osei, Charles Mensah, Gordon A. Awandare, Yaw Aniweh, Lucas N. Amenga-Etego, Gillian Stresman

**Affiliations:** ^1^ Department of Epidemiology, College of Public Health, University of South Florida, Tampa, Florida, USA;; ^2^ Center for Immunization and Infection Research in Cancer (CIIR), H. Lee Moffitt Cancer Center and Research Institute, Tampa, Florida, USA;; ^3^ West African Centre for Cell Biology of Infectious Pathogens, University of Ghana, Accra, Ghana;; ^4^ University of Nebraska Medical Center, Omaha, Nebraska, USA;; ^5^ Department of Biochemistry, Cell and Molecular Biology, University of Ghana, Accra, Ghana

## Abstract

Urban malaria transmission in sub-Saharan Africa remains heterogeneous and incompletely characterized, particularly in rapidly expanding metropolitan areas. We investigated predictors of *Plasmodium falciparum* infection and effect modification of key interventions across the Greater Accra Region of Ghana. Three cross-sectional household cluster visits were conducted during pre-rains, rains, and post-rains periods of the 2024 transmission season. Participants age ≥6 months were tested using multispecies rapid diagnostic tests, microscopy, and loop-mediated isothermal amplification confirmations. Structured administered questionnaires captured sociodemographic, travel, and vector**-**control information. Multilevel logistic regression models estimated adjusted odds ratios (aORs) with 95% CIs. Among 10,716 sampled participants from 3,904 households, *P. falciparum* prevalence ranged from 6.5% to 12% across visit rounds. Although older groups and males had higher odds of infection, participants in the highest socioeconomic status demographic had lower odds. Coverage of indoor residual spraying (IRS) and use of other vector-control methods, including bed nets, mosquito coils, mosquito spray, mosquito repellent, and burning leaves, was low, with no protective association observed for any method. However, interaction associations of bed net use and IRS coverage with malaria infection differed by residency (bed net aOR: 0.49, 95% CI: 0.29–0.86; IRS aORs: 0.59, 95% CI: 0.36–0.97), though stratified models demonstrated mixed findings, with no protective association for either method in either residency. Travel history was not associated with infection. Urban malaria risk in Greater Accra appears to be driven primarily by localized transmission dynamics with spatial heterogeneity in intervention effectiveness, underscoring the need for geographically targeted control strategies.

## INTRODUCTION

Malaria remains a major public health challenge in Africa, accounting for 94% of all cases and 95% of deaths worldwide in 2025.^1^ Although rural areas bear the highest burden in most malaria-endemic countries, urban settings not only sustain malaria transmission but have also experienced rising transmission since at least 2003, making them a vital landscape to achieve malaria elimination.^2^^,^^3^ This burden is expected to continue to increase as urbanization accelerates; by 2030, the 10 countries with the highest malaria burden in sub-Saharan Africa are expected to achieve 50% urbanization, an increase of 119 million urban residents.^2^ These trends underscore the need to adapt malaria response to urban contexts.^2^

A better understanding of urban malaria epidemiology is required to tailor appropriate intervention strategies to the urban context, where transmission differs from rural counterparts. During the 20th century, improvements in economic development, housing construction, and prompt diagnosis and treatment have led to a decrease in urban malaria incidence; though these low levels of transmission have also lowered immunity, there has been an increase in susceptibility to severe disease.^2^^,^^4^^,^^5^ In addition, whereas vector-control strategies such as indoor residual spraying (IRS) and bed nets are among the most effective ways to reduce malaria transmission, they remain underused and their effectiveness comparatively under-studied in urban settings.^2^^,^^6^ Emerging threats such as *Anopheles stephensi*, a vector highly adapted to urban environments that is tolerant of high temperatures and resistant to many common insecticides, can also pose a significant challenge to elimination efforts where it has become established as an important vector.^7^ Human mobility may also complicate urban transmission dynamics because travel between high- and low-transmission settings may facilitate parasite spread and have been associated with increased odds of infection.^2^^,^^8^^,^^9^

Furthermore, identifying which population groups are most at risk of malaria infection and which vector-control strategies are most useful in different urban contexts are vital for designing maximally effective interventions. However, critical knowledge gaps remain regarding the use of vector-control measures and malaria infection and how these vary across time and space.^10^ Moreover, many studies rely on rapid diagnostic tests (RDTs), which are commonly used for point-of-care testing but may be unable to detect low-density and subpatent infections.^11^ As low-density infections contribute to ongoing transmission, diagnostics with enhanced sensitivity are needed to determine effective interventions and characterize overall malaria burden in urban settings.^12^ In addition, although rural travel has been linked to urban malaria, it is unknown whether this holds true across different populations and how risk factors impact these associations.^13^ This study aimed to 1) assess the impact of different vector-control methods on malaria infection; 2) examine how seasonality, travel history, and residency influence this association; and 3) identify populations most at risk to inform a more targeted intervention approach.

## MATERIALS AND METHODS

### Study area.

This study was conducted in the Greater Accra Region of Ghana, West Africa, an area characterized by perennial malaria transmission with seasonal peaks varying by region.^14^ Over half of Ghana’s population already live in urban areas, yet the Greater Accra Region has the lowest bed net ownership and usage nationwide (48.8% and 19.9%, respectively) and a median malaria prevalence of 26 per 1,000 people from 2015 to 2018, highlighting the growing importance of urban malaria control in this area.^14^^–^^17^ Peak malaria transmission periods in the Greater Accra Region typically occur between June and July, following the highest levels of precipitation in May and June.^18^^,^^19^ The predominant *Plasmodium* species responsible for malaria is *Plasmodium falciparum*, accounting for the majority of cases in Ghana, followed by *P. malariae* and *P. ovale;* however, the detection of *An. stephensi*, first reported in 2022, may also introduce new trends.^20^^–^^22^

### Study procedures.

We conducted three household cluster visits from March 2024 to November 2024. The visits were conducted across three periods corresponding with different malaria transmission seasons: March 2, 2024 to April 5, 2024 (Pre-Rains), June 3, 2024 to July 3, 2024 (Rains), and October 7, 2024 to November 2, 2024 (Post-Rains). Eligible health facilities, consisting of registered private, mission, and Ghana Health Service (GHS) facilities, were randomly selected from a list of 436 facilities in the Greater Accra Region that reported to the District Health Information Management Systems (DHIMS). For each visit, a new random cluster of 13 health facilities were randomly selected using spatial stratification by high and low/medium malaria risk levels, as defined by cases reported in the previous year, with intentional oversampling of facilities located in high-risk areas to improve the power to detect infections. For each selected health facility, 100 households within a 750-m radius were selected using a spatial sampling frame, and all members age 6 months or older were invited to participate. If a selected structure were not eligible or household members were not available, the next nearest structure or household was selected. Due to the repeated random selection of health facilities and households for each cluster visit round, a given facility and household may have been selected more than once in all three visits. However, a comparison of household GPS coordinates across survey rounds identified no households located in the same location.

During visits, consenting individuals were administered a structured questionnaire related to both household- and individual-level factors, including materials used in housing construction, wealth indicators, receipt of IRS and use of other vector-control methods, and history of recent travel, using a Samsung Android tablet device with the Survey Solution suite, release 25.01.2.^23^ Completed data forms were securely transferred via a direct internet connection to a 256-bit SSL-encrypted, firewall-protected server hosted at the West African Centre for Cell Biology of Infectious Pathogens and accessible to University of South Florida study personnel. After completing the questionnaires, each consenting individual had their axillary temperature measured and a blood sample taken to test for hematopoietic indicators and malaria infection. Specifically, a finger-prick blood sample was used to perform a malaria RDT with the OnSite™ Malaria Pf/*Pan* Ag Rapid Test CE (CTK Biotech, Poway, CA, USA). A thick and thin blood smear on a slide was used to quantify parasite densities by microscopy and four blood spots of 50 *µ*L each on Whatman^®^ 3MM filter paper were collected to test for *P. falciparum* infections using a SYBR Green I based loop-mediated isothermal amplification (LAMP) assay.^24^

### Variables of interest.

The primary outcome for this analysis was a positive *P. falciparum* LAMP test result. Although RDT and microscopy results were also available, LAMP results were chosen due to their increased sensitivity for detecting low-density infections. Covariates of interest included binary indicators for receipt of IRS in the past 12 months and use of other vector-control methods, e.g., having slept under a bed net the previous night and use of mosquito coils, mosquito sprays, mosquito repellent ointments/creams, or burning leaves in the previous week. Season, residency, and travel history were evaluated as both potential effect modifiers and confounders of the association between vector-control use and *P. falciparum* infection. The season was defined by visit rounds conducted in March, June, and October 2024, corresponding to the pre-rain, rain, and post-rain periods, respectively. Residency was defined as urban versus peri-urban based on location.^25^^–^^27^ Specifically, urban areas were classified in alignment with the Ghana Statistical Service definition based on a minimum population size threshold of 5,000 and attributes including physical development, housing, land use dynamics, socioeconomic activities, and governance, whereas peri-urban areas were considered as transitional areas, with a blend of urban characteristics and rural features.^26^^–^^28^ Travel history indicated spending at least one night outside the Greater Accra Region in the previous 4 weeks.

Potential confounders were considered for model inclusion based on epidemiological knowledge of factors that may influence both exposure status and risk of malaria infection. These confounders included the highest level of education attained by the household head, age, sex, number of individuals residing in the dwelling, household size, occupation classified according to ISCO-08, and a measure of socioeconomic status (SES).^29^ This SES measure was derived by categorizing households into wealth quintiles via a principal components analysis of 16 household indicators. Indicators included ownership of livestock; selected household assets; housing construction materials for walls, roofing, and flooring; the degree to which windows had glass; whether eaves were open or closed; the main source of drinking water; and the type of sanitation facilities used. This approach allowed household conditions to be incorporated into the SES classification.

## STATISTICAL ANALYSES

All consenting participants were included in the analysis. Descriptive statistics were generated to summarize participant characteristics and use of vector-control methods by season. Oversampling was accounted for via sampling weights based on the inverse probability of facility selection with raw numeric counts presented along with weighted prevalence percentages or weighted medians representative of the Greater Accra population, as applicable. Variation across seasons was assessed using Pearson’s χ^2^ test for categorical variables and Kruskal–Wallis rank-sum test for continuous variables.

To assess factors associated with *P. falciparum* positivity, generalized linear mixed-effect models with a binomial distribution and logit link were used.^30^ Oversampling of higher transmission areas was accounted for in regression models using sampling weights based on the inverse probability of facility selection and scaled to the population. Random intercepts for both the household and facility were fit to account for clustering at both levels after assessment of intraclass correlation coefficient (ICC) via the latent threshold approach and using likelihood ratio tests to determine substantial clustering.^31^ Model estimation was performed using the glmer function in the lme4 package (R v. 4.5.1; The R Foundation for Statistical Computing, Vienna, Austria).^32^^,^^33^ Both univariable and multivariable models were explored; the multivariable model selection used a forward stepwise selection approach using likelihood ratio tests and Akaike information criterion to assess model fit though a fully adjusted model is also reported for comparison. Potential overlap between health facility catchment areas was evaluated using a 750-m buffer and, where overlap was identified, fully adjusted mixed-effect models excluding overlapping facilities were compared with the primary analysis.

Effect modification was evaluated for each vector-control method, regardless of whether it was included in the final multivariable model. Interaction terms were added to models to evaluate multiplicative interaction, and models with statistically significant interaction terms were further examined using stratified models. Subsequent sensitivity analyses evaluated potential additive interaction through relative excess risk due to interaction, attributable proportions (AP), and synergy index comparisons using Wald 95% confidence intervals. For these sensitivity analyses, odds ratios were used as approximations for risk ratios and comparison stratum were selected based on observed effect estimate directionality from main effect models.

Burning leaves was found to be an uncommon exposure, and additional sensitivity analyses were conducted to assess the robustness of the adjusted effect estimates. First, the exposure’s correlation with all other covariates was examined using Cramer’s V tests, followed by an assessment of clustering at both the facility and household levels. Based on these results, a fully adjusted fixed effects model with facility as a categorical covariate was fit, and the effect estimates were compared with those from the fully adjusted multilevel model. Last, due to the rarity of the exposure, a Firth logistic regression with a penalized likelihood was also compared as an additional sensitivity analysis.^34^ Subsequently, interaction models were considered adequate for assessment if cross-tabulations showed sufficient values available in all cells.

## RESULTS

A total of 10,716 participants from 3,904 households were included for analysis in 36 health facility catchment areas within 39 clusters across the three visit rounds; two facilities were selected twice, and one was selected in all three cluster visits. Participants were predominantly female (56%–62%), with a wide age range, though a relatively consistent proportion of children ≤5 years of age (7.8%–8.8%). Most had some formal education (85%–96%) and reported head of household employment (88%–97%), though a higher proportion of individuals in the high SES group were reported in the final visit round (37% versus 12%–14%) (Table 1).

**Table 1:** Characteristics by season

Characteristic	Pre-Rains *N* = 2,898*	Rains *N* = 3,609*	Post-Rains *N* = 4,209*	*P*-Value^†^
*P. falciparum* Infection	188 (5.6%)	425 (12%)	316 (6.4%)	<0.001
RDT Result (Positive)	63 (1.8%)	88 (2.1%)	75 (1.9%)	0.8
Residency (Urban)	748 (43%)	1,127 (56%)	1,613 (36%)	0.7
Age	28 (0–101)	28 (0–100)	29 (0–94)	0.7
Age (≤5 years)	274 (8.8%)	330 (8.4%)	345 (7.8%)	0.5
Age (Categories)	–	–	–	0.021
≤5	274 (8.8%)	330 (8.4%)	345 (7.8%)	–
6–15	493 (19%)	573 (15%)	518 (12%)	–
16–25	578 (18%)	776 (21%)	868 (21%)	–
26–35	537 (17%)	739 (21%)	920 (23%)	–
36–45	438 (15%)	569 (16%)	739 (18%)	–
>45	578 (22%)	622 (19%)	819 (18%)	–
Sex (female)	1,795 (62%)	2,119 (61%)	2,454 (56%)	0.053
Education (any level)	2,722 (96%)	3,414 (95%)	3,460 (85%)	<0.001
Occupation (any indicated)	2,531 (88%)	3,409 (94%)	4,058 (97%)	<0.001
SES (high)	293 (12%)	572 (14%)	1,461 (37%)	<0.001
Travel	187 (6.5%)	153 (5.1%)	83 (2.2%)	0.055
Number of Households in Dwelling	1.00 (1.00–20.00)	2.00 (1.00–15.00)	1.00 (1.00–10.00)	<0.001
Household Size	3 (1–20)	3 (1–13)	3 (2–6)	<0.001
IRS (past 12 months)	474 (19%)	190 (4.3%)	1,252 (30%)	0.013
Bed Net Use (last night)	281 (8.6%)	368 (9.6%)	713 (18%)	<0.001
Mosquito Coil Use (last week)	1,501 (50%)	1,833 (52%)	2,416 (54%)	0.7
Mosquito Spray Use (last week)	1,511 (54%)	2,206 (57%)	2,727 (65%)	0.3
Mosquito Repellent Use (last week)	184 (7.1%)	80 (2.8%)	707 (21%)	<0.001
Burning Leaves (last week)	12 (0.7%)	42 (1.8%)	130 (3.0%)	0.084

IRS = indoor residual spraying; SES = socioeconomic status.

* *n* (unweighted) (%); Weighted median (min–max).

^†^ Survey-weighted Pearson’s χ^2^ test for categorical variables and survey-weighted Kruskal–Wallis test for continuous variables.

Overall, *P. falciparum* positivity ranged between 5.6% and 12% by season, peaking during the rainy season. Across seasons, coverage of IRS and use of other vector-control methods were generally low. The most frequently used methods were mosquito coils and mosquito spray, reported by 50% to 54% and 54% to 65% of participants by season, respectively. Coverage of IRS ranged from 4.3% to 30% by season, and use of bed nets ranged from 8.6% to 18%; use of mosquito repellents ranged from 2.8% to 21%, and burning leaves from 0.7% to 3.0%. Coverage of IRS and use of other vector-control methods were highest in the post-rain season (Table 1). Across cluster visit rounds, only a small percentage of participants reported travel outside of the Greater Accra Region within the past 4 weeks, ranging from 2.2% to 6.5% by season.

Assessment of random effects using latent threshold ICC estimation and likelihood ratio tests (LRTs) revealed significant clustering at both the household and facility level (ICC: 0.170 and 0.071, respectively; LRT *p* <0.001 for addition of household and *P* <0.001 for addition of facility); random effects for each were included in all models. That is, approximately 17% and 7% of variance in LAMP positivity are attributable to differences between households and facilities, respectively. In addition, two facility pairs in the pre-rain and rainy periods had overlapping 750-m buffer areas, whereas no overlaps were identified during the post-rain period. A comparison of the primary analysis model with fully adjusted models fit excluding overlapping facilities revealed comparable effect estimates, with no changes in magnitude, direction, or statistical significance.

Variables retained in the multivariable model included age group, sex, SES quintile, and season (Table 2). Sensitivity analyses using a fully adjusted model, inclusive of all potential confounders, revealed similar effect estimates to those from the forward stepwise-selected models and overall conclusions were unchanged (Supplemental Table 1). Older age groups (versus participants ≤5 years of age) had significantly increased odds of *P. falciparum* infection ranging from 1.65 (95% CI: 1.14–2.40) for 26- to 35-year-olds to 2.13 (95% CI: 1.47–3.07) for those over 45 years of age. Females had lower odds of infection than males (aOR: 0.80; 95% CI: 0.68–0.93) and those in both the middle and highest SES quintiles had decreased odds in comparison with the lowest SES quintile (middle SES aOR: 0.74; 95% CI: 0.55–0.98; highest SES aOR: 0.62; 95% CI: 0.47–0.82). The rainy season was also associated with increased odds of infection (aOR: 1.76; 95% CI: 1.17–2.63).

**Table 2:** Univariable and multivariable fixed effect estimates

Predictor	Odds Ratio	95% CI	*P*-Value	Odds Ratio	95% CI	*P*-Value
Age, ≤5 years	Reference			Reference		
Age, 6–15 years	**1.92**	**(1.32, 2.81)**	**0.001**	**1.95**	**(1.33, 2.85)**	**0.001**
Age, 16–25 years	**1.89**	**(1.32, 2.73)**	**0.001**	**1.98**	**(1.37, 2.85)**	**< 0.001**
Age, 26–35 years	**1.57**	**(1.09, 2.27)**	**0.016**	**1.65**	**(1.14, 2.4)**	**0.008**
Age, 36–45 years	**1.75**	**(1.21, 2.55)**	**0.003**	**1.87**	**(1.28, 2.72)**	**0.001**
Age, >45 years	**2.02**	**(1.4, 2.91)**	**< 0.001**	**2.13**	**(1.47, 3.07)**	**< 0.001**
Sex, Female	**0.83**	**(0.7, 0.97)**	**0.017**	**0.80**	**(0.68, 0.93)**	**0.005**
SES Quintile, Lowest	Reference			Reference		
SES Quintile, Low	0.88	(0.68, 1.12)	0.297	0.86	(0.67, 1.11)	0.255
SES Quintile, Middle	**0.75**	**(0.56, 0.99)**	**0.044**	**0.74**	**(0.55, 0.98)**	**0.036**
SES Quintile, High	0.84	(0.65, 1.1)	0.203	0.82	(0.63, 1.07)	0.153
SES Quintile, Highest	**0.64**	**(0.49, 0.84)**	**0.002**	**0.62**	**(0.47, 0.82)**	**0.001**
IRS (last 12 months)	0.97	(0.76, 1.24)	0.823	–	–	–
Bed Net Use (last night)	1.08	(0.84, 1.38)	0.546	–	–	–
Mosquito Coils (last week)	0.95	(0.8, 1.12)	0.535	–	–	–
Mosquito Spray (last week)	0.95	(0.8, 1.12)	0.520	–	–	–
Mosquito Repellent (last week)	1.13	(0.84, 1.54)	0.418	–	–	–
Burning Leaves (last week)	1.70	(0.98, 2.94)	0.057	**1.83**	**(1.05, 3.19)**	**0.032**
Travel	1.00	(0.69, 1.46)	0.991	–	–	–
Season, Pre-Rains	Reference			Reference		
Season, Rains	**1.75**	**(1.16, 2.63)**	**0.007**	**1.76**	**(1.17, 2.63)**	**0.006**
Season, Post-Rains	1.04	(0.71, 1.53)	0.825	1.05	(0.72, 1.55)	0.786
Residency, Urban	0.89	(0.58, 1.37)	0.601	–	–	–

CI = confidence interval; SES = socioeconomic status.

Bold values indicate statistically significant results (*P* < 0.05).

No vector-control methods were associated with lower odds of malaria infection in any of the evaluated models. Among the vector-control methods examined, burning leaves was retained in the multivariable model. Burning leaves was associated with increased odds of infection (aOR: 1.83; 95% CI: 1.05–3.19). Because burning leaves was found to be an uncommon exposure, several sensitivity analyses were undertaken as previously described. Burning leaves was found to share little correlation with other covariates of interest, and a multivariable fixed effects model with facility included demonstrated a similar odds ratio as the final multivariable model (aOR = 1.77; 95% CI: 1.14–2.75) as did a Firth logistic regression (aOR = 1.79; 95% CI: 1.13–2.72).

Each vector-control method was also evaluated for each of the potential effect modifiers (Tables 3–5; Supplemental Tables 2–14). Evidence of multiplicative interaction by residence was observed for IRS coverage, bed net use, and mosquito repellent use (IRS aOR: 0.55; 95% CI: 0.32–0.95; bed net aOR: 0.55; 95% CI: 0.32–0.95; mosquito repellent aOR: 0.53, 95% CI: 0.29–0.99). Subsequent stratified models revealed no association between bed net use and LAMP positivity in urban areas (aOR = 0.61; 95% CI: 0.34–1.07) or peri-urban areas (aOR = 1.29; 95% CI: 0.98–1.69). In contrast, IRS coverage showed increased odds with peri-urban residence (aOR: 1.34; 95% CI: 1.03–1.75) and no association in urban areas (aOR: 0.77; 95% CI: 0.42–1.42). Similarly, mosquito repellent use was associated with increased odds in peri-urban areas (aOR: 1.70; 95% CI: 1.19–2.41) and no association in urban areas (aOR: 0.89; 95% CI: 0.46–1.71). No other multiplicative interaction associations were observed. Subsequent sensitivity analyses evaluating interaction on the additive scale largely agreed with the multiplicative results, indicating differences by residency for IRS coverage, bed net use, and mosquito repellent use. In contrast, although no multiplicative interaction was observed for burning leaves by residency, a significant attributable proportion was found on the additive scale (AP: 0.63; 95% CI: 0.25–1.02).

**Table 3:** Multivariable fixed-effects estimates with IRS coverage interaction effects for season, travel, and residency*

Predictor	Season			Travel			Residency		
	Odds Ratio	95% Ci	*P*-Value	Odds Ratio	95% Ci	*P*-Value	Odds Ratio	95% CI	*P*-Value
IRS (last 12 months)	1.01	(0.59, 1.72)	0.977	1.11	(0.86, 1.44)	0.414	**1.34**	**(1.00, 1.81)**	**0.050**
Season, Pre-Rains	Reference			–	–	–	–	–	–
Season, Rains	**1.75**	**(1.15, 2.65)**	**0.009**	–	–	–	–	–	–
Season, Post-Rains	1.04	(0.7, 1.57)	0.835	–			–	–	–
IRS (last 12 months)* Season, Rains	1.11	(0.5, 2.48)	0.798	–	–	–	–	–	–
IRS (last 12 months)* Season, Post-Rains	1.17	(0.62, 2.18)	0.631	–	–	–	–	–	–
Travel	–	–	–	0.85	(0.55, 1.31)	0.456	–	–	–
IRS (last 12 months)* Travel	–	–	–	1.41	(0.43, 4.68)	0.573	–	–	–
Residency, Urban	–	–	–	–	–	–	0.96	(0.66, 1.38)	0.817
IRS (last 12 months)* Residency, Urban	–	–	–	–	–	–	**0.55**	**(0.32, 0.95)**	**0.031**

IRS = indoor residual spraying; SES = socioeconomic status.

* Model adjusted for age (categorical), sex, season, and SES quintiles. Output for all fixed effect estimates available in Supplemental Table 2.

Bold values indicate statistically significant results (*P* < 0.05).

**Table 4:** Multivariable fixed-effects estimates with bed net use interaction effects for season, travel, and residency*

Predictor	Season			Travel			Residency		
	Odds Ratio	95% CI	*P*-Value	Odds Ratio	95% CI	*P*-Value	Odds Ratio	95% CI	*P*-Value
Bed Net Use (last night)	1.55	(0.89, 2.68)	0.121	1.06	(0.82, 1.37)	0.683	1.32	(0.98, 1.79)	0.072
Season, Pre-Rains	Reference			–	–	–	–	–	–
Season, Rains	**1.87**	–	**0.003**	–	–	–	–		
Season, Post-Rains	1.12	–	0.57	–			–	–	–
Bed Net Use (last night)* Season, Rains	0.62	–	0.182	–	–	–	–	–	–
Bed net use (last night)* Season, Post-Rains	0.66	–	0.228	–	–	–	–	–	–
Travel	–	–	–	0.88	–	–	–	–	–
Bed Net Use (last night)* Travel	–	–	–	1.77	–	–	–	–	–
Residency, Urban	–	–	–	–	–	–	0.93	(0.65, 1.33)	0.693
Bed Net Use (last night)* Residency, Urban	–	–	–	–	–	–	**0.55**	**(0.32, 0.95)**	**0.032**

CI = confidence interval.

* Model adjusted for age (categorical), sex, season, and SES quintiles. Output for all fixed effect estimates available in Supplemental Table 3.

Bold values indicate statistically significant results (*P* < 0.05).

**Table 5:** Multivariable fixed-effects estimates with mosquito repellents use interaction effects for season, travel, and residency*

Predictor	Season			Travel			Residency		
	Odds Ratio	95% CI	*P*-Value	Odds Ratio	95% CI	*P*-Value	Odds Ratio	95% CI	*P*-Value
Mosquito Repellent Use (last week)	1.03	(0.48, 2.21)	0.936	1.28	(0.94, 1.76)	0.119	**1.65**	**(1.1, 2.48)**	**0.015**
Season, Pre-Rains	Reference			Reference			Reference		
Season, Rains	**1.76**	**(1.17, 2.65)**	**0.007**	**1.79**	**(1.19, 2.69)**	**0.005**	**1.80**	**(1.19, 2.72)**	**0.006**
Season, Post-Rains	1.03	(0.7, 1.53)	0.873	1.06	(0.72, 1.56)	0.767	1.06	(0.71, 1.58)	0.766
Mosquito Repellent Use (last week)* Season, Rains	1.14	(0.39, 3.34)	0.817	–	–	–	–	–	–
Mosquito Repellent Use (last week)* Season, Post-Rains	1.29	(0.55, 3.02)	0.557	–	–	–	–	–	–
Travel	–	–	–	1.00	(0.68, 1.49)	0.981	–	–	–
Mosquito Repellent Use (last week)* Travel	–	–	–	0.36	(0.05, 2.54)	0.303	–	–	–
Residency, Urban	–	–	–	–	–	–	0.92	(0.63, 1.34)	0.658
Mosquito Repellent Use (last week)* Residency, Urban	–	–	–	–	–	–	**0.53**	**(0.29, 0.99)**	**0.047**

CI = confidence interval; SES = socioeconomic status.

* Model adjusted for age (categorical), sex, season, and SES quintiles. Output for all fixed effect estimates available in Supplemental Table 6.

Bold values indicate statistically significant results (*P* < 0.05).

## DISCUSSION

In this study, we present demographic and epidemiological trends in *P. falciparum* infections in an urban setting, incorporating vector-control methods, travel history, seasonality, and residency in randomly selected health facility catchment areas of Ghana’s Greater Accra Region. Overall, these results align with existing research, demonstrating low infection prevalence overall, low bed net use, increased odds of malaria in individuals older than 5 years, and seasonal variation consistent with low-transmission settings.^2^^,^^17^^,^^35^ However, there were several important findings including a lack of association for travel or residency, differing associations of bed net use by residency, and increased odds of infection among those reporting burning leaves as a vector-control method.

### Travel.

Population mobility is thought to be a key driver of urban malaria transmission whereby continuous importation facilitates sustained transmission.^2^^,^^9^^,^^13^^,^^36^ However, the overall prevalence of travel in the current study was relatively low, and no association between travel and *P. falciparum* infection was observed across models, suggesting travel outside the Greater Accra Region may contribute relatively little to the overall burden of malaria in this study population. Although recent travel outside the Greater Accra Region may not capture all relevant forms of travel, including daily commuting or seasonal migration, overnight travel was intentionally selected because it aligns with peak *Anopheles* activity, suggesting mobility may play a lesser role in this population. In addition, emerging evidence suggests irrigated urban agriculture may be a primary driver of urban malaria transmission in Greater Accra, and these same environments have also been linked to the spread of *An. stephensi*, a vector particularly well-suited to urban environments.^37^^,^^38^ These factors are of particular importance, given recent predictive modeling which suggests the Greater Accra Region is well-suited to *An. stephensi*, raising concerns it may become an increasing threat.^39^ Taken together, these findings suggest mobility alone may not be the sole factor driving malaria transmission in the Greater Accra Region, and evolving ecological drivers may play a substantial role.

### Vector-control methods.

In the current study, overall vector-control coverage and reported use was low, consistent with previous reports in the Greater Accra Region and urban African environments.^2^^,^^14^^,^^16^^,^^17^ Low coverage alone can diminish the overall population-level effect of these interventions, and this factor likely contributed to the lack of protective association observed. High levels of pyrethroid target site resistance and other metabolic resistance mechanisms in the major malaria vector, *An. gambiae* s.l., may have contributed to the observed diminished impact of vector-control methods.^40^^,^^41^ Specifically, a recent study of vector distribution in Accra found the L1014F allele, a mutation associated with pyrethroid and DDT resistance, present in 100% of *An. gambiae* s.l., apart from *An. gambiae* s.s., which had 90% frequency.^42^ Taken together, these factors highlight the broader challenge of vector-control methods in urban African environments where their protection may be undermined by low usage and growing insecticide resistance.

An additional consideration is the potential for confounding by indication. Previous research has shown that perceived severity of malaria infection is associated with intended use of preventive measures.^43^ Given the cross-sectional nature of this study, vector-control measures were not randomly assigned, and it is possible individuals may be more likely to use vector-control measures in response to a higher perceived mosquito density or perceived malaria risk. Therefore, the absence of an observed protective association for these vector-control measures should be interpreted cautiously, because the observed associations may reflect a combination of true limitations in effectiveness, such as low coverage and insecticide resistance, and residual confounding from unmeasured differences in perceived malaria risk.

Although no overall association between IRS coverage, bed net use, or mosquito repellents and malaria infection was detected, interaction analyses suggested the relationship of these interventions may differ by area. This finding supports existing evidence that malaria transmission in urban environments is highly heterogeneous, often driven by focal breeding sites.^2^^,^^3^^,^^44^ As a result, the impact of vector-control methods may vary substantially across urban geographic areas, and subregional mapping and tailoring of interventions are recommended for effective operational planning.^2^ This study’s findings therefore complement these recommendations and support the need to consider differences between urban and peri-urban areas as part of intervention deployment strategies.

Although burning leaves as a vector-control method was reported infrequently, this practice was consistently associated with increased *P. falciparum* prevalence across models, and subsequent sensitivity analyses supported the robustness of these findings. Although this vector-control method has not been widely researched, this finding contrasts with the limited existing research indicating a protective association of burning certain plant matter as vector control.^45^ One potential explanation is that individuals who burn leaves may also spend more time outdoors, thereby increasing their exposure, though additional research is needed to examine this. This exposure group may represent a population with elevated risk and could be considered for additional prevention strategies in future.

### Seasonality.

Although malaria transmission in urban environments largely exhibits seasonal variation, rainfall alone may not fully explain these fluctuations.^22^^,^^46^^,^^47^ Recent evidence has suggested temperature and land use may play a larger role than rainfall in predicting seasonal variation.^47^ In addition, evidence from the Greater Accra Region suggests irrigated urban agriculture is a primary driver of transmission, wheras a separate study in Ghana found malaria prevalence to be driven more by topography and proximity to bodies of water and waterways than rainfall itself.^14^^,^^37^ However, an additional study of aggregated district-level malaria incidence in the Greater Accra Region supported a seasonal trend that aligned with rainfall patterns.^18^ Taken together, the seasonality observed in the current study is consistent with prior evidence but may reflect the combined influence of rainfall and other ecological and environmental factors that temporally align. As urban environments become increasingly relevant to malaria control, identifying the factors underlying seasonal transmission may reveal important targets for future interventions.

### Limitations.

This study had several limitations. First, the cross-sectional nature of this study limits causal interpretation. Because all exposure and outcome information were collected at a single visit, the temporal ordering cannot be established with full certainty. Observed associations may therefore reflect residual confounding by underlying transmission risk, behavioral adaption to previous infections, or other unmeasured factors. In addition, seasonal comparisons should be interpreted with caution because independent samples were selected for each survey round. As a result, although health facility catchment areas were randomly selected, it cannot be excluded that observed seasonal differences may reflect a combination of seasonal transmission differences and differences within the sampled populations and geographic areas. However, this analysis remains informative for investigating epidemiological trends and generating hypotheses that can be further studied in future longitudinal studies.

Second, because all exposures and covariates, apart from the outcome of interest, were self-reported, recall bias cannot be ruled out. Vector-control methods and travel history, which rely on recent, time-bound behaviors, may have been prone to recall bias misclassification, resulting in nondifferential, attenuated effect estimates. However, apart from IRS coverage, which was indicated within the past 12 months, recall periods were limited to the previous 1 to 4 weeks, lessening the likelihood of substantial recall bias.

Third, the comparatively small number of positive cases observed, although encouraging, may have masked the impact of travel and vector-control methods on *P. falciparum* infection. Similarly, the association between burning leaves and *P. falciparum* infection may also be affected by the comparatively low number of individuals reporting this exposure.

Finally, although the random selection approach of included health facility areas and the robustness of subsequent sensitivity analyses lend strength to these findings, it is possible that the small overall number of included facility areas and households sampled may not reflect the true effect of these exposures in this densely populated region. As such, additional research on a larger scale that allows for examination of these less common exposures is warranted.

## CONCLUSION

This study’s findings reinforce the importance of adapting malaria prevention efforts to the realities of urban transmission. Although population mobility is widely recognized as a key driver of malaria transmission in urban areas, this study found that malaria burden in Greater Accra is likely associated with local transmission and varies across the region. This finding supports the idea that, in settings with comparatively low mobility, other ecological and entomological factors may be more influential. Similarly, IRS coverage and use of other vector-control methods is low overall in urban settings, and their relative efficacy may be affected by context-dependent factors. Therefore, further research is needed to better understand which vector-control methods are appropriate for malaria control in urban settings. Although seasonality played a role in urban malaria transmission, the underlying factors contributing to seasonality warrant further investigation, because they may represent important targets for future control efforts. As urbanization expands in malaria-endemic countries, this study adds to the growing evidence on the heterogeneity of urban malaria transmission. These findings may assist further research and development of tailored, evidence-based interventions for other African cities in high-burden countries.

## Supplemental Materials

10.4269/ajtmh.26-0325 Supplemental Materials
