# Supplementary material for: Epidemiology of Urban Malaria in the Greater Accra Region of Ghana: Predictors and Effect Modification across a Transmission Season
Source: Am J Trop Med Hyg. 2026 Oct 27:tpmd260325. Online ahead of print. doi: 10.4269/ajtmh.26-0325 (PMC13770338; doi:10.4269/ajtmh.26-0325)
Supplement: Supplementary file 1 [file tpmd260325.SD1.pdf]

Supplemental Tables

Supplemental Table 1. Fully adjusted multivariable fixed effect estimates, inclusive of all potential confounders

Predictor	Odds Ratio	95% CI	p-value
Age, <=5		Reference	
Age, 6-15	**2.02**	**(1.36, 2.99)**	**< 0.001**
Age, 16-25	**2.04**	**(1.4, 2.99)**	**< 0.001**
Age, 26-35	**1.72**	**(1.17, 2.53)**	**0.005**
Age, 36-45	**1.92**	**(1.29, 2.85)**	**0.001**
Age, >45	**2.34**	**(1.6, 3.44)**	**< 0.001**
Sex, Female	**0.80**	**(0.68, 0.94)**	**0.009**
Number of HHs in dwelling	1.00	(0.95, 1.05)	0.956
SES quintile, Lowest		Reference	
SES quintile, Low	0.85	(0.65, 1.11)	0.228
SES quintile, Middle	0.75	(0.55, 1.01)	0.054
SES quintile, High	0.79	(0.59, 1.05)	0.104
SES quintile, Highest	**0.57**	**(0.42, 0.77)**	**< 0.001**
Education (Any level)	0.93	(0.67, 1.3)	0.673
Occupation (Any indicated)	0.99	(0.69, 1.41)	0.958
Household size	1.01	(0.95, 1.08)	0.761
IRS (last 12 months)	1.04	(0.8, 1.37)	0.755
Bednet use (last night)	1.10	(0.85, 1.42)	0.485
Mosquito coils use (last week)	0.96	(0.8, 1.15)	0.658
Mosquito spray use (last week)	0.98	(0.82, 1.18)	0.845
Mosquito repellent use (last week)	1.21	(0.87, 1.69)	0.256
Burning leaves (last week)	**1.82**	**(1.03, 3.21)**	**0.038**
Travel	0.88	(0.59, 1.33)	0.553
Season, Pre-Rains		Reference	
Season, Rains	**1.81**	**(1.2, 2.73)**	**0.005**
Season, Post-Rains	1.08	(0.72, 1.6)	0.718
Residency, Urban	0.84	(0.59, 1.2)	0.341

Bold values indicate statistically significant results ($P < .05$).

Supplemental Table 2. Multivariable fixed effects estimates with IRS coverage interaction effects for season, travel, and residency

	SEASON			TRAVEL			RESIDENCY		
**Predictor**	**Odds Ratio**	**95% CI**	**p-value**	**Odds Ratio**	**95% CI**	**p-value**	**Odds Ratio**	**95% CI**	**p-value**
Age, <= 5		Reference			Reference			Reference	
Age, 6-15	**1.99**	**(1.35, 2.95)**	**0.001**	**2.03**	**(1.37, 3.0)**	**<0.001**	**1.99**	**(1.35, 2.94)**	**0.001**
Age, 16-25	**2.02**	**(1.39, 2.94)**	**<0.001**	**2.05**	**(1.41, 3.0)**	**<0.001**	**2.01**	**(1.38, 2.93)**	**<0.001**
Age, 26-35	**1.71**	**(1.17, 2.49)**	**0.006**	**1.74**	**(1.19, 2.55)**	**0.004**	**1.70**	**(1.16, 2.48)**	**0.006**
Age, 36-45	**1.86**	**(1.26, 2.75)**	**0.002**	**1.90**	**(1.28, 2.81)**	**0.001**	**1.86**	**(1.26, 2.75)**	**0.002**
Age, >45	**2.27**	**(1.56, 3.31)**	**<0.001**	**2.32**	**(1.59, 3.4)**	**<0.001**	**2.27**	**(1.56, 3.31)**	**<0.001**
Sex, Female	**0.80**	**(0.68, 0.95)**	**0.008**	**0.80**	**(0.68, 0.94)**	**0.007**	**0.81**	**(0.68, 0.95)**	**0.009**
SES quintile, Lowest		Reference			Reference			Reference	
SES quintile, Low	0.84	(0.65, 1.08)	0.177	0.84	(0.65, 1.09)	0.19	0.84	(0.65, 1.08)	0.174
SES quintile, Middle	**0.73**	**(0.55, 0.98)**	**0.037**	**0.73**	**(0.55, 0.98)**	**0.036**	**0.73**	**(0.54, 0.98)**	**0.033**
SES quintile, High	0.79	(0.6, 1.04)	0.09	0.79	(0.6, 1.04)	0.092	0.79	(0.6, 1.04)	0.09
SES quintile, Highest	**0.59**	**(0.44, 0.79)**	**<0.001**	**0.59**	**(0.44, 0.79)**	**<0.001**	**0.60**	**(0.45, 0.79)**	**<0.001**
IRS (last 12 months)	1.01	(0.59, 1.72)	0.977	1.11	(0.86, 1.44)	0.414	**1.34**	**(1, 1.81)**	**0.05**
Season, Pre-Rains		Reference			Reference			Reference	
Season, Rains	1.75	(1.15, 2.65)	0.009	**1.77**	**(1.17, 2.67)**	**0.006**	**1.77**	**(1.18, 2.66)**	**0.006**
Season, Post-Rains	1.04	(0.7, 1.57)	0.835	1.07	(0.73, 1.58)	0.728	1.06	(0.72, 1.56)	0.774
IRS * Season, Rains	1.11	(0.5, 2.48)	0.798						
IRS * Season, Post-Rains	1.17	(0.62, 2.18)	0.631						
Travel				0.85	(0.55, 1.31)	0.456			
IRS (last 12 months) * Travel				1.41	(0.43, 4.68)	0.573			
Residency, Urban							0.96	(0.66, 1.38)	0.817
IRS * Residency, Urban							**0.55**	**(0.32, 0.95)**	**0.031**

Bold values indicate statistically significant results (*P* < .05).

Supplemental Table 3. Multivariable fixed effects with bednet use interaction effects for season, travel, and residency

	SEASON			TRAVEL			RESIDENCY		
**Predictor**	**Odds Ratio**	**95% CI**	**p-value**	**Odds Ratio**	**95% CI**	**p-value**	**Odds Ratio**	**95% CI**	**p-value**
Age, <=5		Reference			Reference			Reference	
Age, 6-15	**1.95**	**(1.33, 2.85)**	**0.001**	**1.98**	**(1.35, 2.9)**	**<0.001**	**1.96**	**(1.34, 2.86)**	**0.001**
Age, 16-25	**1.97**	**(1.36, 2.84)**	**<0.001**	**2.00**	**(1.38, 2.89)**	**<0.001**	**1.96**	**(1.36, 2.83)**	**<0.001**
Age, 26-35	**1.65**	**(1.14, 2.39)**	**0.008**	**1.68**	**(1.16, 2.44)**	**0.006**	**1.64**	**(1.13, 2.38)**	**0.009**
Age, 36-45	**1.87**	**(1.28, 2.72)**	**0.001**	**1.89**	**(1.29, 2.77)**	**0.001**	**1.86**	**(1.27, 2.71)**	**0.001**
Age, >45	**2.12**	**(1.47, 3.07)**	**<0.001**	**2.17**	**(1.49, 3.14)**	**<0.001**	**2.12**	**(1.46, 3.06)**	**<0.001**
Sex, Female	**0.80**	**(0.68, 0.94)**	**0.006**	**0.80**	**(0.68, 0.94)**	**0.006**	**0.80**	**(0.68, 0.94)**	**0.007**
SES quintile, Lowest		Reference			Reference			Reference	
SES quintile, Low	0.86	(0.67, 1.11)	0.245	0.86	(0.67, 1.11)	0.244	0.85	(0.66, 1.1)	0.216
SES quintile, Middle	**0.73**	**(0.55, 0.98)**	**0.034**	**0.73**	**(0.55, 0.97)**	**0.032**	**0.73**	**(0.55, 0.98)**	**0.034**
SES quintile, High	0.82	(0.63, 1.07)	0.151	0.82	(0.63, 1.07)	0.14	0.81	(0.62, 1.06)	0.131
SES quintile, Highest	**0.63**	**(0.47, 0.83)**	**0.001**	**0.62**	**(0.47, 0.82)**	**0.001**	**0.62**	**(0.47, 0.82)**	**0.001**
Bednet use (last night)	1.55	(0.89, 2.68)	0.121	1.06	(0.82, 1.37)	0.683	1.32	(0.98, 1.79)	0.072
Season, Pre-Rains		Reference			Reference			Reference	
Season, Rains	**1.87**	**(1.24, 2.81)**	**0.003**	**1.78**	**(1.19, 2.66)**	**0.005**	**1.82**	**(1.22, 2.72)**	**0.004**
Season, Post-Rains	1.12	(0.76, 1.66)	0.570	1.07	(0.73, 1.57)	0.737	1.09	(0.74, 1.61)	0.647
Bednet use (last night) * Season, Rains	0.62	(0.31, 1.25)	0.182						
Bednet use (last night) * Season, Post-Rains	0.66	(0.34, 1.29)	0.228						
Travel				0.88	(0.58, 1.34)	0.552			
Bednet use (last night) * Travel				1.77	(0.62, 5.08)	0.287			
Residency, Urban							0.93	(0.65, 1.33)	0.693
Bednet use (last night) * Residency, Urban							**0.55**	**(0.32, 0.95)**	**0.032**

Bold values indicate statistically significant results ($P < .05$).

Supplemental Table 4. Multivariable fixed effects estimates with mosquito coil use interaction effects for season, travel, and residency

	SEASON			TRAVEL			RESIDENCY		
**Predictor**	**Odds Ratio**	**95% CI**	**p-value**	**Odds Ratio**	**95% CI**	**p-value**	**Odds Ratio**	**95% CI**	**p-value**
Age, <= 5		Reference			Reference			Reference	
Age, 6-15	**1.94**	**(1.33, 2.84)**	**0.001**	**1.98**	**(1.35, 2.9)**	**<0.001**	**1.94**	**(1.33, 2.84)**	**0.001**
Age, 16-25	**1.96**	**(1.36, 2.83)**	**<0.001**	**2.00**	**(1.38, 2.89)**	**<0.001**	**1.96**	**(1.36, 2.83)**	**<0.001**
Age, 26-35	**1.65**	**(1.14, 2.38)**	**0.008**	**1.68**	**(1.15, 2.43)**	**0.007**	**1.64**	**(1.14, 2.38)**	**0.008**
Age, 36-45	**1.86**	**(1.27, 2.71)**	**0.001**	**1.89**	**(1.29, 2.77)**	**0.001**	**1.86**	**(1.27, 2.71)**	**0.001**
Age, >45	**2.11**	**(1.46, 3.05)**	**<0.001**	**2.16**	**(1.49, 3.14)**	**<0.001**	**2.11**	**(1.46, 3.06)**	**<0.001**
Sex, Female	**0.80**	**(0.68, 0.94)**	**0.006**	**0.80**	**(0.68, 0.93)**	**0.005**	**0.80**	**(0.68, 0.94)**	**0.006**
SES quintile, Lowest		Reference			Reference			Reference	
SES quintile, Low	0.85	(0.66, 1.1)	0.22	0.86	(0.66, 1.1)	0.231	0.85	(0.66, 1.1)	0.224
SES quintile, Middle	**0.73**	**(0.55, 0.97)**	**0.032**	**0.73**	**(0.55, 0.98)**	**0.034**	**0.73**	**(0.55, 0.98)**	**0.035**
SES quintile, High	0.81	(0.62, 1.06)	0.13	0.81	(0.62, 1.06)	0.131	0.81	(0.62, 1.06)	0.121
SES quintile, Highest	**0.62**	**(0.47, 0.83)**	**0.001**	**0.62**	**(0.47, 0.82)**	**0.001**	**0.62**	**(0.47, 0.83)**	**0.001**
Mosquito coils use (last week)	0.94	(0.65, 1.35)	0.725	0.98	(0.82, 1.17)	0.828	0.94	(0.75, 1.18)	0.595
Season, Pre-Rains		Reference			Reference			Reference	
Season, Rains	**1.72**	**(1.09, 2.73)**	**0.02**	**1.78**	**(1.19, 2.66)**	**0.005**	**1.81**	**(1.21, 2.71)**	**0.004**
Season, Post-Rains	1.04	(0.66, 1.63)	0.873	1.07	(0.73, 1.58)	0.715	1.10	(0.75, 1.62)	0.631
Mosquito coils use (last week) * Season, Rains	1.06	(0.68, 1.65)	0.797						
Mosquito coils use (last week) * Season, Post-Rains	1.06	(0.67, 1.7)	0.794						
Travel				0.92	(0.54, 1.54)	0.738			
Mosquito coils use (last week) * Travel				1.09	(0.51, 2.32)	0.827			
Residency, Urban							0.82	(0.55, 1.22)	0.327
Mosquito coils use (last week) * Residency, Urban							1.11	(0.78, 1.57)	0.562

Bold values indicate statistically significant results (*P* < .05).

Supplemental Table 5. Multivariable fixed effects estimates with mosquito spray use interaction effects for season, travel, and residency

	SEASON			TRAVEL			RESIDENCY		
**Predictor**	**Odds Ratio**	**95% CI**	**p-value**	**Odds Ratio**	**95% CI**	**p-value**	**Odds Ratio**	**95% CI**	**p-value**
Age, <= 5		Reference			Reference			Reference	
Age, 6-15	**1.94**	**(1.32, 2.83)**	**0.001**	**1.98**	**(1.35, 2.9)**	**<0.001**	**1.95**	**(1.33, 2.85)**	**0.001**
Age, 16-25	**1.96**	**(1.36, 2.83)**	**<0.001**	**2.00**	**(1.39, 2.9)**	**<0.001**	**1.96**	**(1.36, 2.83)**	**<0.001**
Age, 26-35	**1.64**	**(1.14, 2.38)**	**0.008**	**1.68**	**(1.16, 2.43)**	**0.007**	**1.65**	**(1.14, 2.39)**	**0.008**
Age, 36-45	**1.86**	**(1.27, 2.71)**	**0.001**	**1.90**	**(1.3, 2.79)**	**0.001**	**1.86**	**(1.28, 2.72)**	**0.001**
Age, >45	**2.11**	**(1.46, 3.06)**	**<0.001**	**2.17**	**(1.49, 3.14)**	**<0.001**	**2.12**	**(1.47, 3.07)**	**<0.001**
Sex, Female	**0.80**	**(0.68, 0.94)**	**0.006**	**0.79**	**(0.68, 0.93)**	**0.005**	**0.80**	**(0.68, 0.94)**	**0.006**
SES quintile, Lowest		Reference			Reference			Reference	
SES quintile, Low	0.85	(0.66, 1.1)	0.221	0.85	(0.66, 1.1)	0.224	0.85	(0.66, 1.1)	0.207
SES quintile, Middle	**0.73**	**(0.55, 0.98)**	**0.034**	**0.73**	**(0.55, 0.98)**	**0.037**	**0.74**	**(0.55, 0.98)**	**0.038**
SES quintile, High	0.82	(0.62, 1.07)	0.139	0.81	(0.62, 1.06)	0.133	0.81	(0.62, 1.06)	0.123
SES quintile, Highest	**0.62**	**(0.47, 0.83)**	**0.001**	**0.62**	**(0.46, 0.82)**	**0.001**	**0.62**	**(0.47, 0.83)**	**0.001**
Mosquito spray use (last week)	0.94	(0.64, 1.36)	0.727	1.03	(0.86, 1.23)	0.785	1.10	(0.87, 1.38)	0.43
Season, Pre-Rains		Reference			Reference			Reference	
Season, Rains	**1.72**	**(1.07, 2.76)**	**0.025**	**1.78**	**(1.2, 2.67)**	**0.005**	**1.81**	**(1.21, 2.7)**	**0.004**
Season, Post-Rains	1.00	(0.63, 1.6)	0.984	1.07	(0.73, 1.58)	0.713	1.10	(0.75, 1.62)	0.622
Mosquito spray use (last week) * Season, Rains	1.07	(0.68, 1.68)	0.784						
Mosquito spray use (last week) * Season, Post-Rains	1.12	(0.69, 1.82)	0.641						
Travel				1.26	(0.73, 2.18)	0.397			
Mosquito spray use (last week) * Travel				0.59	(0.28, 1.26)	0.175			
Residency, Urban							0.99	(0.66, 1.49)	0.959
Mosquito spray use (last week) * Residency, Urban							0.79	(0.56, 1.13)	0.203

Bold values indicate statistically significant results (*P* < .05).

Supplemental Table 6. Multivariable fixed effects estimates with mosquito repellents use interaction effects for season, travel, and residency

	SEASON			TRAVEL			RESIDENCY		
**Predictor**	**Odds Ratio**	**95% CI**	**p-value**	**Odds Ratio**	**95% CI**	**p-value**	**Odds Ratio**	**95% CI**	**p-value**
Age, <= 5		Reference			Reference			Reference	
Age, 6-15	**1.94**	**(1.33, 2.84)**	**0.001**	**1.99**	**(1.36, 2.91)**	**<0.001**	**1.93**	**(1.32, 2.83)**	**0.001**
Age, 16-25	**1.96**	**(1.36, 2.84)**	**<0.001**	**2.01**	**(1.39, 2.9)**	**<0.001**	**1.97**	**(1.36, 2.84)**	**<0.001**
Age, 26-35	**1.65**	**(1.14, 2.39)**	**0.008**	**1.69**	**(1.16, 2.45)**	**0.006**	**1.64**	**(1.13, 2.38)**	**0.009**
Age, 36-45	**1.86**	**(1.28, 2.72)**	**0.001**	**1.91**	**(1.3, 2.79)**	**0.001**	**1.86**	**(1.28, 2.72)**	**0.001**
Age, >45	**2.12**	**(1.47, 3.07)**	**<0.001**	**2.18**	**(1.5, 3.16)**	**<0.001**	**2.12**	**(1.46, 3.06)**	**<0.001**
Sex, Female	**0.80**	**(0.68, 0.94)**	**0.006**	**0.79**	**(0.68, 0.93)**	**0.005**	**0.80**	**(0.68, 0.94)**	**0.006**
SES quintile, Lowest		Reference			Reference			Reference	
SES quintile, Low	0.85	(0.66, 1.1)	0.221	0.86	(0.66, 1.11)	0.238	0.86	(0.67, 1.11)	0.235
SES quintile, Middle	**0.73**	**(0.55, 0.98)**	**0.034**	**0.73**	**(0.55, 0.97)**	**0.033**	**0.73**	**(0.55, 0.97)**	**0.031**
SES quintile, High	0.82	(0.63, 1.06)	0.131	0.81	(0.62, 1.06)	0.131	0.82	(0.63, 1.07)	0.139
SES quintile, Highest	**0.61**	**(0.46, 0.8)**	**0.001**	**0.60**	**(0.46, 0.8)**	**<0.001**	**0.61**	**(0.46, 0.81)**	**0.001**
Mosquito repellent use (last week)	1.03	(0.48, 2.21)	0.936	1.28	(0.94, 1.76)	0.119	**1.65**	**(1.1, 2.48)**	**0.015**
Season, Pre-Rains		Reference			Reference			Reference	
Season, Rains	**1.76**	**(1.17, 2.65)**	**0.007**	**1.79**	**(1.19, 2.69)**	**0.005**	**1.80**	**(1.19, 2.72)**	**0.006**
Season, Post-Rains	1.03	(0.7, 1.53)	0.873	1.06	(0.72, 1.56)	0.767	1.06	(0.71, 1.58)	0.766
Mosquito repellent use * Season, Rains	1.14	(0.39, 3.34)	0.817						
Mosquito repellent use * Season, Post-Rains	1.29	(0.55, 3.02)	0.557						
Travel				1.00	(0.68, 1.49)	0.981			
Mosquito repellent use (last week) * Travel				0.36	(0.05, 2.54)	0.303			
Residency, Urban							0.92	(0.63, 1.34)	0.658
Mosquito repellent use (last week) * Residency, Urban							**0.53**	**(0.29, 0.99)**	**0.047**

Bold values indicate statistically significant results (*P* < .05).

Supplemental Table 7. Multivariable fixed effects estimates with burning leaves use interaction effects for residency*

	RESIDENCY		
**Predictor**	**Odds Ratio**	**95% CI**	**p-value**
Age, <= 5		Reference	
Age, 6-15	**1.95**	**(1.33, 2.85)**	**0.001**
Age, 16-25	**1.97**	**(1.36, 2.84)**	**<0.001**
Age, 26-35	**1.65**	**(1.14, 2.38)**	**0.008**
Age, 36-45	**1.86**	**(1.27, 2.72)**	**0.001**
Age, >45	**2.12**	**(1.46, 3.06)**	**<0.001**
Sex, Female	**0.80**	**(0.68, 0.93)**	**0.005**
SES quintile, Lowest		Reference	
SES quintile, Low	0.87	(0.67, 1.12)	0.268
SES quintile, Middle	**0.74**	**(0.56, 0.99)**	**0.04**
SES quintile, High	0.83	(0.63, 1.08)	0.162
SES quintile, Highest	**0.63**	**(0.47, 0.83)**	**0.001**
Burning leaves (last week)	**3.25**	**(1.44, 7.37)**	**0.005**
Season, Pre-Rains		Reference	
Season, Rains	**1.78**	**(1.18, 2.67)**	**0.006**
Season, Post-Rains	1.07	(0.73, 1.58)	0.726
Residency, Urban	0.88	(0.61, 1.26)	0.485
Burning leaves (last week) * Residency, Urban	0.37	(0.12, 1.14)	0.084

*Note: Insufficient observations were available for interaction analysis between burning leaves and either season or travel history.

Bold values indicate statistically significant results (*P* < .05).

Supplemental Table 8. Multivariable fixed effects estimates including IRS, stratified to peri-urban residence

Predictor	Odds Ratio	95% CI	p-value
Age, <= 5		Reference	
Age, 6-15	1.52	(0.98, 2.36)	0.061
Age, 16-25	1.51	(0.99, 2.3)	0.053
Age, 26-35	1.30	(0.85, 1.99)	0.227
Age, 36-45	1.35	(0.87, 2.11)	0.182
Age, >45	**1.68**	**(1.1, 2.58)**	**0.017**
Sex, Female	0.90	(0.74, 1.09)	0.273
SES quintile, Lowest		Reference	
SES quintile, Low	0.88	(0.65, 1.19)	0.391
SES quintile, Middle	0.71	(0.5, 1.02)	0.062
SES quintile, High	0.86	(0.64, 1.17)	0.336
SES quintile, Highest	**0.65**	**(0.47, 0.9)**	**0.009**
Season, Pre-Rains		Reference	
Season, Rains	1.24	(0.77, 1.99)	0.383
Season, Post-Rains	0.82	(0.52, 1.3)	0.395
IRS (last 12 months)	**1.34**	**(1.03, 1.75)**	**0.028**

Bold values indicate statistically significant results ($P < .05$).

Supplemental Table 9. Multivariable fixed effects estimates including IRS, stratified to urban residence*

Predictor	Odds Ratio	95% CI	p-value
Age, <=5		Reference	
Age, 6-15	**4.34**	**(1.94, 9.69)**	**<0.001**
Age, 16-25	**3.66**	**(1.66, 8.06)**	**0.001**
Age, 26-35	**3.46**	**(1.58, 7.57)**	**0.002**
Age, 36-45	**3.73**	**(1.69, 8.27)**	**0.001**
Age, >45	**4.96**	**(2.27, 10.83)**	**<0.001**
Sex, Female	**0.63**	**(0.46, 0.84)**	**0.002**
SES quintile, Lowest		Reference	
SES quintile, Low	0.73	(0.41, 1.29)	0.282
SES quintile, Middle	0.64	(0.35, 1.18)	0.156
SES quintile, High	0.66	(0.34, 1.31)	0.234
SES quintile, Highest	**0.50**	**(0.26, 0.96)**	**0.036**
Season, Pre-Rains		Reference	
Season, Rains	**5.80**	**(3.16, 10.63)**	**<0.001**
Season, Post-Rains	**3.29**	**(1.75, 6.18)**	**<0.001**
IRS (last 12 months)	0.77	(0.42, 1.42)	0.408

*Note: facility-level intercept omitted due to negligible facility-level variation found after adjustment.

Bold values indicate statistically significant results (*P* < .05).

Supplemental Table 10. Multivariable fixed effects estimates including bednets, stratified to peri-urban residence

Predictor	Odds Ratio	95% CI	p-value
Age, <= 5		Reference	
Age, 6-15	1.53	(1, 2.36)	0.052
Age, 16-25	1.47	(0.97, 2.22)	0.068
Age, 26-35	1.29	(0.85, 1.97)	0.227
Age, 36-45	1.30	(0.84, 2.02)	0.233
Age, >45	**1.62**	**(1.06, 2.47)**	**0.025**
Sex, Female	0.89	(0.74, 1.09)	0.259
SES quintile, Lowest		Reference	
SES quintile, Low	0.90	(0.67, 1.21)	0.486
SES quintile, Middle	0.74	(0.52, 1.05)	0.096
SES quintile, High	0.91	(0.68, 1.22)	0.539
SES quintile, Highest	**0.70**	**(0.51, 0.96)**	**0.028**

Predictor	Odds Ratio	95% CI	p-value
Season, Pre-Rains		Reference	
Season, Rains	1.23	(0.77, 1.97)	0.391
Season, Post-Rains	0.85	(0.54, 1.34)	0.485
Bednet (last night)	1.29	(0.98, 1.69)	0.07

Bold values indicate statistically significant results (*P* < .05).

Supplemental Table 11. Multivariable fixed effects estimates including bednets, stratified to urban residence

Predictor	Odds Ratio	95% CI	p-value
Age, <= 5		Reference	
Age, 6-15	**3.97**	**(1.83, 8.59)**	**<0.001**
Age, 16-25	**3.47**	**(1.63, 7.39)**	**0.001**
Age, 26-35	**3.01**	**(1.42, 6.39)**	**0.004**
Age, 36-45	**3.72**	**(1.74, 7.95)**	**0.001**
Age, >45	**4.20**	**(1.98, 8.88)**	**<0.001**
Sex, Female	**0.63**	**(0.47, 0.84)**	**0.002**
SES quintile, Lowest		Reference	
SES quintile, Low	0.72	(0.41, 1.27)	0.259
SES quintile, Middle	0.61	(0.33, 1.1)	0.101
SES quintile, High	0.65	(0.34, 1.26)	0.206
SES quintile, Highest	**0.50**	**(0.27, 0.93)**	**0.029**
Season, Pre-Rains		Reference	
Season, Rains	**6.34**	**(3.55, 11.34)**	**<0.001**
Season, Post-Rains	**3.42**	**(1.83, 6.41)**	**<0.001**
Bednet (last night)	0.61	(0.34, 1.07)	0.086

Bold values indicate statistically significant results (*P* < .05).

Supplemental Table 12. Multivariable fixed effects estimates including mosquito repellents, stratified to peri-urban residence

Predictor	Odds Ratio	95% CI	p-value
Age, <= 5		Reference	
Age, 6-15	1.51	(0.98, 2.32)	0.061
Age, 16-25	1.47	(0.98, 2.23)	0.065
Age, 26-35	1.30	(0.85, 1.97)	0.225
Age, 36-45	1.31	(0.85, 2.03)	0.223

Predictor	Odds Ratio	95% CI	p-value
Age, >45	**1.61**	**(1.06, 2.46)**	**0.027**
Sex, Female	0.89	(0.73, 1.07)	0.22
SES quintile, Lowest		Reference	
SES quintile, Low	0.90	(0.67, 1.22)	0.51
SES quintile, Middle	0.73	(0.51, 1.03)	0.077
SES quintile, High	0.91	(0.68, 1.23)	0.543
SES quintile, Highest	**0.67**	**(0.48, 0.92)**	**0.013**
Season, Pre-Rains		Reference	
Season, Rains	1.23	(0.76, 2)	0.397
Season, Post-Rains	0.84	(0.53, 1.34)	0.461
Mosquito repellent (last week)	**1.70**	**(1.19, 2.41)**	**0.003**

Bold values indicate statistically significant results (*P* < .05).

Supplemental Table 13. Multivariable fixed effects estimates including mosquito repellents, stratified to urban residence

Predictor	Odds Ratio	95% CI	p-value
Age, <= 5		Reference	
Age, 6-15	**3.94**	**(1.82, 8.5)**	**<0.001**
Age, 16-25	**3.47**	**(1.63, 7.38)**	**0.001**
Age, 26-35	**3.03**	**(1.43, 6.42)**	**0.004**
Age, 36-45	**3.69**	**(1.73, 7.88)**	**0.001**
Age, >45	**4.21**	**(2, 8.9)**	**<0.001**
Sex, Female	**0.63**	**(0.47, 0.84)**	**0.002**
SES quintile, Lowest		Reference	
SES quintile, Low	0.74	(0.42, 1.3)	0.3
SES quintile, Middle	0.61	(0.34, 1.12)	0.111
SES quintile, High	0.67	(0.35, 1.29)	0.228
SES quintile, Highest	**0.52**	**(0.28, 0.97)**	**0.039**
Season, Pre-Rains		Reference	
Season, Rains	**6.23**	**(3.48, 11.15)**	**<0.001**
Season, Post-Rains	**3.28**	**(1.75, 6.14)**	**<0.001**
Mosquito repellent (last week)	0.89	(0.46, 1.71)	0.723

Bold values indicate statistically significant results (*P* < .05).

Supplemental Table 14. Additive interaction results from multivariable fixed effect estimates

Name*	RERI	95% CI	p-value	AP	95% CI	p-value	SI	95% CI	p-value
No use of IRS (last 12 months) * Travel	-0.33	(-1.72, 1.05)	0.637	-0.44	(-2.29, 1.42)	0.643	-2.46	(-39, 34.08)	0.853
No use of IRS (last 12 months) * Season, Rains	-0.20	(-1.52, 1.12)	0.767	-0.12	(-0.85, 0.62)	0.761	0.79	(-0.27, 1.84)	0.691
No use of IRS (last 12 months) * Season, Post-Rains	-0.17	(-0.9, 0.55)	0.639	-0.17	(-0.81, 0.47)	0.610	0.17	(-1.99, 2.33)	0.452
No use of IRS (last 12 months) * Residency, Peri-Urban	-0.85	(-1.85, 0.15)	0.095	**-0.60**	**(-1.14, -0.06)**	**0.030**	**0.33**	**(0.03, 0.63)**	**0.000**
Bednet use (last night) * Travel	0.71	(-0.9, 2.32)	0.386	0.43	(-0.17, 1.03)	0.156	-10.26	(-91.33, 70.82)	0.786
Bednet use (last night) * Season, Rains	-0.61	(-1.75, 0.53)	0.295	-0.34	(-1.06, 0.38)	0.356	0.57	(-0.06, 1.2)	0.181
Bednet use (last night) * Season, Post-Rains	-0.52	(-1.48, 0.44)	0.287	-0.45	(-1.35, 0.45)	0.324	0.22	(-0.57, 1.01)	0.053
Bednet use (last night) * Residency, Peri-Urban	**0.62**	**(0.08, 1.15)**	**0.024**	**0.44**	**(0.13, 0.74)**	**0.006**	-2.13	(-10.21, 5.94)	0.447
No use of Mosquito coils (last week) * Travel	-0.08	(-0.82, 0.65)	0.826	-0.09	(-0.9, 0.73)	0.832	-4.32	(-179.61, 170.96)	0.953
No use of Mosquito coils (last week) * Season, Rains	-0.05	(-0.66, 0.55)	0.861	-0.03	(-0.36, 0.3)	0.861	0.94	(0.3, 1.58)	0.852
No use of Mosquito coils (last week) * Season, Post-Rains	-0.06	(-0.57, 0.44)	0.803	-0.06	(-0.52, 0.4)	0.803	0.62	(-1.46, 2.71)	0.724
No use of Mosquito coils (last week) * Residency, Peri-Urban	0.11	(-0.24, 0.47)	0.542	0.09	(-0.21, 0.4)	0.543	2.87	(-19.25, 24.99)	0.869
No use of Mosquito spray (last week) * Travel	0.51	(-0.26, 1.28)	0.193	0.41	(-0.05, 0.87)	0.079	-0.84	(-3.77, 2.08)	0.217
No use of Mosquito spray (last week) * Season, Rains	-0.06	(-0.69, 0.56)	0.841	-0.04	(-0.37, 0.3)	0.842	0.93	(0.28, 1.58)	0.831
No use of Mosquito spray (last week) * Season, Post-Rains	-0.12	(-0.65, 0.41)	0.652	-0.11	(-0.61, 0.38)	0.651	0.38	(-1.45, 2.2)	0.503
No use of Mosquito spray (last week) * Residency, Peri-Urban	-0.26	(-0.69, 0.17)	0.233	-0.22	(-0.6, 0.15)	0.236	0.38	(-0.37, 1.13)	0.107
Mosquito repellent use (last week) * Travel	-0.83	(-1.86, 0.2)	0.116	-1.80	(-7.26, 3.66)	0.518	-1.87	(-6.77, 3.03)	0.252

Name*	RERI	95% CI	p-value	AP	95% CI	p-value	SI	95% CI	p-value
Mosquito repellent use (last week) * Season, Rains	0.27	(-1.48, 2.03)	0.762	0.13	(-0.63, 0.9)	0.736	1.34	(-1.06, 3.75)	0.781
Mosquito repellent use (last week) * Season, Post-Rains	0.31	(-0.62, 1.24)	0.513	0.23	(-0.42, 0.87)	0.490	5.80	(-73.96, 85.56)	0.906
Mosquito repellent use (last week) * Residency, Peri-Urban	0.83	(-0.01, 1.67)	0.052	**0.46**	**(0.14, 0.78)**	**0.005**	-25.65	(-554, 502.7)	0.921
Burning leaves (last week) * Residency, Peri-Urban	2.35	(-0.89, 5.58)	0.155	**0.63**	**(0.25, 1.02)**	**0.001**	7.65	(-15.18, 30.48)	0.568

*Note: Comparison stratum were selected based on observed effect estimate directionality from main effects models.

Bold values indicate statistically significant results ($P < .05$).
